# Optimizing drone-based pollination method by using efficient target detection and path planning for complex durian orchards

**DOI:** 10.1038/s41598-025-04370-0

**Published:** 2025-07-01

**Authors:** Ruipeng Tang, Jianxun Tang, Mohamad Sofian Abu Talip, Narendra Kumar Aridas, Binghong Guan

**Affiliations:** 1 School of Electrical, Electronic and Mechanical Engineering, University of Bristol, BS8 1UB Bristol, UK; 2 Faculty of Electronics and Electrical Engineering, Zhaoqing University, No. 55, Zhaoqing City, Guangdong Province China; 3 Department of Electrical Engineering, Faculty of Engineering, University of Malaya, 50603 Kuala Lumpur, Malaysia; 4 Faculty of Business and Economics, University of Malaya, 50603 Kuala Lumpur, Malaysia

**Keywords:** Artificial intelligence automates pollination, Drone pollination, Smart durian garden management, Agricultural automation, Agricultural image analysis, Industry, innovation and infrastructure, Computer science, Sustainability, Plant sciences, Mathematics and computing

## Abstract

Durian is a valuable tropical fruit whose pollination heavily relies on bats and nocturnal insects. However, environmental degradation and pesticide usage have reduced insect populations, leading to inefficient natural pollination. This study proposes an AI-powered drone-based pollination method for complex durian orchards, integrating improved object detection and optimized path planning. Specifically, we enhance the YOLO-v8 algorithm using the GhostNet lightweight network to reduce computational complexity while boosting detection precision. For path planning, we develop an Enhanced TSP (EN-TSP) algorithm based on a branch and bound strategy with least-cost optimization. Experimental results demonstrate that the proposed method improves detection accuracy by 5.85% and path efficiency by 26.89% compared to baseline algorithms. The novel use of GhostNet with YOLO-v8 enables superior detection of durian flowers under low-light and occluded conditions, while EN-TSP ensures globally optimal drone routes, reducing travel distance and improving operational reliability. This integrated solution advances smart agriculture by enabling scalable, efficient, and precise pollination, reducing labor costs and increasing durian yield and quality.

## Introduction

Durian, a mainstay of Southeast Asia’s agricultural economy, is facing a severe pollination crisis. Over the past decade, natural pollinators such as bats and nocturnal insects have declined by 40–60% due to habitat loss and pesticide abuse^1^. Artificial pollination is expensive (about $120–150 per acre^2^). Although drone pollination has great potential, it faces two major technical bottlenecks: real-time flower detection in low light and high vegetation density in complex orchard environments and global path planning in dynamic multi-target scenarios^3^. The details are as follows:

### Existing object detection technology and its limitations

In the agricultural field, target detection technology is widely used in the identification and positioning of crop flowers. Cui et al.^4^ proposed an improved Faster R-CNN algorithm, which enhances the detection capability of small targets in complex backgrounds by optimizing the feature pyramid network (FPN) and anchor frame design; it also introduces a context enhancement module, combined with multi-scale feature fusion, to improve the discrimination between targets and backgrounds in drone images. Aucone et al.^5^ proposed a collaborative strategy of morphology and feedback control, which dynamically adjusts the drone morphology to adapt to unknown flexible obstacles; combined with real-time sensor feedback, autonomous obstacle avoidance and path planning are achieved. Janagi et al.^6^ used a lightweight convolutional neural network combined with the optical flow method to achieve real-time moving target detection in dynamic scenes; and introduced an attention mechanism to enhance the ability to distinguish small targets and complex backgrounds. Zhou et al.^7^ optimized the multi-drone task allocation based on an improved particle swarm algorithm, taking into account task priority, energy consumption and collaborative efficiency; it also introduced dynamic inertia weights and constraint processing mechanisms to improve the algorithm convergence speed and global search capabilities. El-Kenawy et al.^8^ proposed the Grey Goose Optimizer (GGO) algorithm inspired by nature. It was tested on 19 datasets from the UCI machine learning repository and solved a series of engineering benchmark functions and case studies. Benyamin et al.^9^ introduced the PO algorithm (Jaguar Optimizer) and novel intelligent mechanism to perform phase change operations during optimization and balance the two phases so that each phase is automatically adjusted according to the nature of the problem. Li et al.^10^ proposed a YOLO-v9 model that combines programmable gradient information (PGI) and generalized efficient layer aggregation network to achieve accurate identification of corn pests and diseases. Liu et al.^11^ proposed a RicePest-DETR model based on Transformer end-to-end detection mechanism to accurately identify small rice pests, which can effectively detect small, densely distributed, and multi-scale rice pests.

Although the above studies have achieved some results, they have some disadvantages. Many algorithms lack the ability to adjust in real time in dynamic environments, especially in scenes with dense vegetation or low light, where detection performance may decline. Although some studies have optimized small target detection, it is difficult to effectively handle multi-target detection problems in complex backgrounds due to the shallow network depth. Some algorithms have high computational complexity and require large computing resources in practical applications, making it difficult to meet real-time requirements. The current research gap is the lack of a target detection algorithm that can efficiently detect small targets in complex environments, meet the computing resource constraints of the UAV platform, and have real-time performance. Although the YOLO-v9 and DETR algorithms demonstrate the potential of the new generation of detection algorithms in small target recognition, feature aggregation, and multi-scale modeling, these algorithm frameworks are still in the early stages of exploration and have not yet been widely tested in diverse, complex backgrounds, and cross-regional datasets. As a result, their performance in real farmland environments is still unstable, and there is a risk of overfitting or insufficient adaptability to unstructured backgrounds. In response to the above research gaps, this paper proposes an improved target detection algorithm (IM-YOLO algorithm) to improve the accuracy and efficiency of target detection in UAV pollination tasks, while reducing computational complexity and meeting real-time requirements.

### Path planning technology and its limitations

The application of drones in agriculture is becoming more and more widespread, and path planning is one of its key technologies. Chen et al.^12^ proposed an intelligent air-ground integrated network architecture called Lasagna to provide intelligent wireless communication, computing and caching services. Lee et al.^13^ proposed a real-time predictive diagnosis framework based on deep learning, which is designed for bedside detection scenarios and realizes rapid pathogen identification and disease classification through lightweight convolutional neural networks. Li et al.^14^ used computer vision to perceive the position of mobile targets in real time and combine programmable metasurfaces to dynamically adjust the phase and amplitude of electromagnetic waves to achieve target tracking and adaptive wireless communication. It also uses reinforcement learning algorithms to coordinate the response of metasurface units, synchronously optimize communication signal strength and target positioning accuracy, and reduce multipath interference. Zhou et al.^15^ proposed a drone anomaly detection method based on wavelet decomposition and stacked denoising autoencoders. It takes into account the negative impact of noisy data and the feature extraction ability of deep learning models, aiming to improve the accuracy of the proposed anomaly detection method by using wavelet decomposition and stacked denoising autoencoder methods. Chen et al.^16^ proposed a fair and efficient MAC protocol based on CSMA/CA, using multi-user MIMO to achieve concurrent uplink transmission from different drones. Wang et al.^17^ proposed a UAV-assisted URLLC scheme for edge users, using information age as an indicator of system latency to achieve the performance requirements of ultra-reliable low-latency communication. Gao et al.^18^ applied model-based curve fitting to obtain modeling parameters based on the theoretical closed-form energy model in existing literature and proposed a theoretical energy model for rotary-wing UAVs. Yin et al.^19^ constructed autonomous navigation of UAVs in a three-dimensional environment with adaptive control as a Markov decision process and proposed a deep reinforcement learning algorithm. They also proposed a new speed constraint loss function and added it to the original actor loss to improve the speed control capability of the UAV.

The above algorithms have made certain progress in their respective fields, but there are still some shortcomings in the path planning of UAV pollination. In dynamic environments and complex backgrounds, many algorithms lack the ability to adjust in real time, which affects the accuracy and efficiency of path planning. Some algorithms have high computational complexity and are difficult to run in real time on resource-constrained UAV platforms, affecting the actual application effect. In complex orchard environments, some algorithms have limited ability to identify and avoid dynamic obstacles, which affects the efficiency and safety of UAV path planning. Therefore, the current research gap lies in the lack of an algorithm that can efficiently and real-time plan UAV pollination paths in complex orchard environments, which can adapt to dynamic obstacles and run under limited computing resources. In response to the above research gaps, this paper proposes an improved path planning algorithm (EN-TSP algorithm) to improve the path planning efficiency and real-time adaptability of UAVs in complex orchard environments, while reducing the computational complexity to meet the actual needs of UAV pollination tasks.

## Materials and methods

### Data collection and processing

#### Data collection

Data collection for this study was conducted in Area C of the durian production base in Pengxiangzhou, Malaysia, focusing on four durian varieties: Musang King, Red Prawn, Sultan King (D24) and XO. Data collection covers two main flowering periods: June to August 2022, and December 2022 to January 2023. During these time periods, durian flowers open and pollinate most frequently, ensuring the representativeness and diversity of the data. In order to obtain high-quality image data, this study installed multiple infrared high-definition cameras in the orchard. The cameras were installed at a height of 2.5 meters and facing the flower area of the durian tree to ensure that the opening and pollination process of the flowers could be fully captured. These cameras have the following parameters:

- Resolution: 1920 × 1080 pixels to ensure image clarity and detail.
- Frame rate: 30 frames per second, suitable for capturing dynamic scenes.
- Field of view: 120 degrees, covering a wider monitoring range.
- Infrared function: Supports night shooting to ensure clear images can be obtained under low light conditions.

**Fig. 1:**
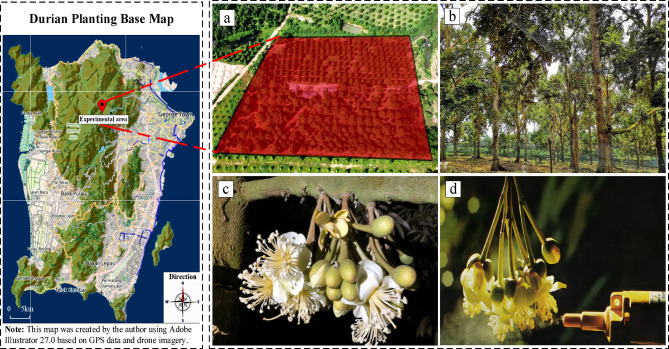
The durian pollination experimental area and drone pollination process.

The data collection time is from 7:00 to 23:00 every day, covering the main opening and pollination periods of durian flowers. The camera automatically captures static images at a frequency of 5 s and transmits them to the central server in real time for storage. During the entire data collection period, a total of about 10,000 original images were collected. Figure 1 shows the durian pollination experimental area and drone pollination process. (This map was created by the author based on field research data using Adobe Illustrator 27.0 software. All visualization elements (including spatial layout and annotation) were manually constructed based on drone aerial images and GPS coordinates collected in Pengerang, Malaysia)

#### Data preprocessing

In the data preprocessing stage, the original images were first screened to remove images with blur, severe occlusion or poor lighting conditions and 7250 qualified durian flower images were obtained. These images were classified according to the flower morphology as follows: 2439 long columnar images, 1945 short columnar images, 1652 bell-shaped images, and 1214 tubular flowers; Subsequently, the dataset was divided into training, validation, and test sets in a ratio of 7:2:1 to ensure the effectiveness and generalization of model training. In order to train the object detection model, the durian flowers in the image need to be accurately annotated. The annotation process is carried out in a semi-automatic manner. The first is preliminary detection. The image is preliminarily detected using the pre-trained object detection model to generate candidate boxes. The next step is manual correction. Professional annotators manually correct the candidate boxes to ensure that the bounding box accurately surrounds each flower and annotates the corresponding flower category. The next step is quality inspection. Random sampling inspection is performed on the annotation results to ensure the consistency and accuracy of the annotation quality. Finally, the standard annotation file (such as XML format) is generated for subsequent model training. 

In order to solve the problem of data imbalance and improve the robustness of the model, a three-point data enhancement strategy is adopted. The first is geometric transformation. First, the random rotation, horizontal and vertical flipping, random cropping and scaling are used to simulate the flower morphology under different perspectives. Then comes the lighting adjustment. By randomly adjusting the brightness, contrast and saturation of the image, different lighting conditions are simulated. Finally, noise is added. By adding Gaussian noise and random occlusion to the image, the model’s adaptability to complex backgrounds and interference is enhanced. Through these strategies, it expands the diversity of training data and improves the generalization ability of the model in different environments.

#### Parameter tuning and algorithm selection

During the model training process, YOLO-v8 was selected as the basic target detection algorithm, and the GhostNet lightweight network was introduced to reduce the computational complexity. It reduces the model parameters by about 30%, while maintaining the detection accuracy and improving the inference speed. The details are as follows:

- Learning rate: Initially set to 0.001, and dynamically adjusted using the cosine annealing strategy.
- Batch size: Set to 16 according to the GPU memory capacity.
- Training rounds: Set to 100 rounds, and monitor the model performance on the validation set, using the early stopping strategy to prevent overfitting.

In the model inference stage, the optimized GhostNet model was tested on the NVIDIA RTX 3080 GPU, with an average inference time of about 12 milliseconds per image, meeting the needs of real-time detection. In addition, the computational complexity (FLOPs) of the model is about 8.5 GFLOPs, and the number of parameters is about 5.8 million (M). While maintaining high accuracy, it achieves low computational overhead and is suitable for deployment on resource-constrained drone platforms.

### Experimental setup

This study uses the TSP scheduling algorithm^20^ to plan the route scheduling sequence of drones between multiple operating areas. It also uses the IM-YOLO algorithm to identify targets in the operating area, which improves the efficient operation of durian pollination targets in multiple areas. In order to verify the performance of these algorithms, this study collects pictures of durian trees, branches, fruits and surrounding environments under different perspectives, different periods and various environmental conditions. It also uses the photo processing software Pix4D to convert 2D photos into 3D models to create a realistic 3D scene. Then, the generated 3D model is supported by Microsoft AirSim^21^, which builds a durian garden simulation environment for drone pollination operations and configures drones and sensors (such as RGB cameras, LiDAR (infrared cameras and laser radar)) to simulate the real pollination tasks. During the entire flight process, the drone collects a depth map every 3 s and extracts targets based on four depth maps. The target information vector is fused according to the relative position relationship of drones to obtain the target information representation at the current moment. It combines with the target information at the previous moment to represent the calculated navigation route. Finally, the drone’s pollination task in the 3D scene is tested. The simulation data is analyzed to optimize the drone’s pollination efficiency and path planning algorithm.

In the model training process of this study, the hardware is trained on a computer equipped with NVIDIA GeForce RTX 3080 GPU, Intel Core i9-10900K CPU and 64GB RAM. The software is the operating system Ubuntu 20.04 LTS, and the main libraries used include Python 3.8, PyTorch 1.9.0, CUDA 11.1 and cuDNN 8.1. The training parameters are set as follows:

- Batch size: set to 4, that is, 4 samples are processed per training iteration.
- Training rounds: set to 50, that is, the entire dataset will be traversed 50 times.
- Optimizer: The RMSprop algorithm is used to update the model weights, the initial learning rate is set to 0.003, and the learning rate decay is 0.0001.
- Activation function: The SELU (Scaled Exponential Linear Unit) function is used to improve the nonlinear expression ability of the model.
- Classifier: After feature extraction, XGBoost classifier is used for the final classification task.

Through the above configuration, the model is trained with optimized parameter settings in an efficient hardware environment, thereby improving the performance and accuracy of object detection.

### Improved TSP scheduling algorithm

In view of the high computational complexity and easy fall into the local optimal solution of the traditional TSP algorithm in large-scale path planning problems, this study introduces the branch and bound method based on minimum cost as an improvement strategy. This has the following advantages:

- By constructing a state space tree and pruning the search nodes based on the cost lower bound function, the need for exhaustive calculation of paths is greatly reduced.
- Using the cost matrix reduction method, the lower bound estimate of the optimal solution can be quickly obtained, and redundant paths can be effectively avoided during the iteration process.
- Compared with heuristic algorithms, this method has more theoretical guarantees in terms of global optimality and is especially suitable for path scheduling scenarios in irregular agricultural areas with densely distributed targets.

**Fig. 2:**
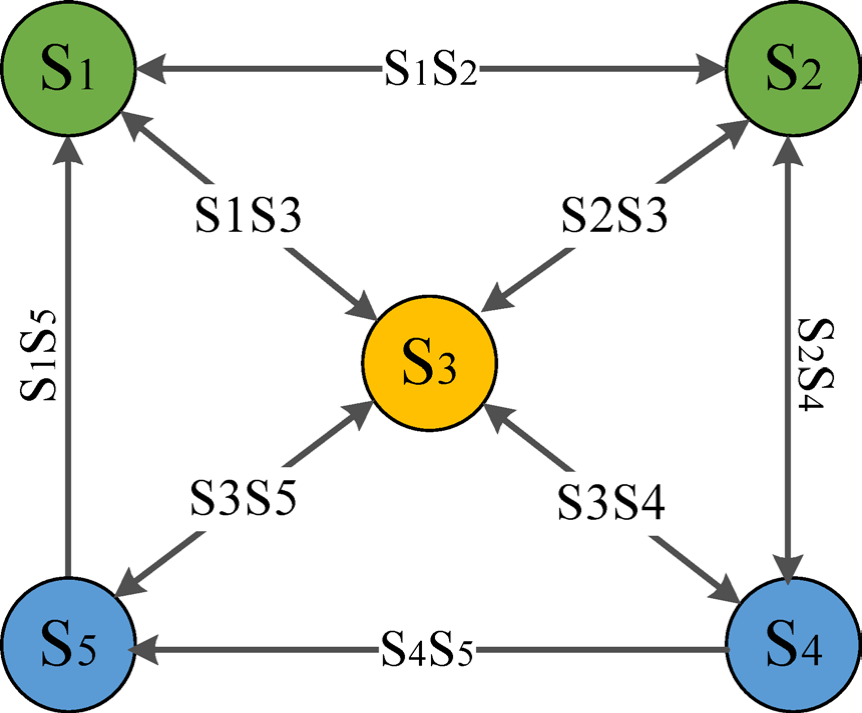
The structure of Bidirectional TSP algorithm.

After the above comparison, this study comprehensively considers calculation accuracy, globality and the ability to adapt to complex path networks. Before the drone carries out the pollination operations in multiple areas, it needs to plan the routes for multiple operations, so this study uses the TSP scheduling algorithm to find the shortest scheduling route and return to the starting point after the operation is completed. The operation endpoint ${\text{S}}_{2}$ of any area $\text{S}$ is selected as the starting point. After traversing other operation areas, only the operation area $\text{S}$ is left, so it returns to ${\text{S}}_{1}$. After completing the operation in the area $\text{S}$, it returns to ${\text{S}}_{2}$, and the overall scheduling route constitutes the Hamilton loop^22^. So this study approximates the drone multi-objective pollination operation as the travelling salesman problem. Unlike the traditional TSP algorithm, the round-trip costs between any two areas in this cruise network are different. So it proposes an improved bidirectional TSP algorithm, which is expressed by the graph theory as follows: If each region is regarded as the node, the multiple regions as a whole constitute a weighted graph, is denoted as $Q=(\text{G},\text{ S})$, $\text{G}$ is the vertex set $G=\{{\text{s}}_{1},{\text{s}}_{2},{\text{s}}_{3},{\text{s}}_{4},\dots ,\text{i}\}$, $\text{S}$ is the edge set, ${\text{d}}_{x,y}$ is the weight on the edge $<\text{x},\text{y}>$, which represents the distance between area $\text{x}$ and $\text{y}$. $\text{i}$ is the number of operation areas, which makes the pollination operation network into the directed graph, as shown in Fig. 2.

In order to find the Hamilton cycle with the minimum weight, the objective function of this network makes the constraint condition as the different cost of round trip between regions. Each area can only be entered and exited once; the starting point of the Hamilton circuit should be selected as the end point of the starting area, which is shown in Formula (1-3):

1 $${\text{d}}_{min}={\sum }_{x=1}^{i}{\sum }_{y=1}^{i}{\text{V}}_{xy}{\text{U}}_{xy}$$

2 $${\text{d}}_{start}={\sum }_{y=1}^{i}{\text{U}}_{xy}=1\forall x\in G$$

3 $${\text{d}}_{end}={\sum }_{x=1}^{i}{\text{U}}_{xy}=1\forall y\in G$$

In Formulas (3–5), ${\text{U}}_{xy}$ represents whether the arc from region to region is selected. Formula (3) is the objective function, which means the minimizing scheduling cost; Formulas (4 and 5) show that each area has the fixed starting and end point, which must pass through one time. The TSP algorithm aims to find the minimum total path length. In the multi-region job scheduling, the round-trip costs between different regions are different, which increases the complexity of problems. So this study uses the branch and bound method based on the least cost to construct the bounding condition^23^, which expands the potential nodes, avoids traversing all possible paths and reduces the computational complexity of the TSP algorithm. The upper bound of bound functions is estimated by the greedy method; the method for solving the lower bound of the objective function value is the method that needs to introduce the concept of reduction matrix and use the reduction matrix to calculate the cost of the TSP problem.

First, the directed graph of the TSP problem is constructed as the cost matrix (adjacency matrix). The purposed data in the figure is only used for the demonstration. The data L of the $i-th$ row and $\text{x}-\text{th}$ column indicates that the cost from the $x-th$ to $y-th$ node is $\text{W}$. The cost of nodes is recorded as $\infty$. For each row and column of the cost matrix, all elements in the row and column are subtracted from the minimum element value, so that there is at least one zero in each row and column, and the resulting matrix becomes the reduction matrix. The sum of the minimum element values in each row and column that are subtracted is called the reduction number $\text{L}$. The reduction matrix of the TSP root node is shown in Formula (4):

4 $$\left(\begin{array}{ccc}\begin{array}{c}\\ {\text{s}}_{1}\end{array}& \begin{array}{c}{\text{s}}_{1}\\ \infty \end{array}& \begin{array}{c}{\text{s}}_{2}\\ 2\end{array}\\ {\text{s}}_{2}& 1& \infty \\ \begin{array}{c}{\text{s}}_{3}\\ {\text{s}}_{4}\\ {\text{s}}_{5}\end{array}& \begin{array}{c}5\\ 5\\ 4\end{array}& \begin{array}{c}4\\ 2\\ 2\end{array}\end{array} \begin{array}{ccc}\begin{array}{c}{\text{s}}_{3}\\ 1\end{array}& \begin{array}{c}{\text{s}}_{4}\\ 3\end{array}& \begin{array}{c}\begin{array}{cc}{\text{s}}_{5}& \text{L}\end{array}\\ \begin{array}{cc}4& -1\end{array}\end{array}\\ 4& 4& \begin{array}{cc}2& -1\end{array}\\ \begin{array}{c}\infty \\ 2\\ 4\end{array}& \begin{array}{c}2\\ \infty \\ 2\end{array}& \begin{array}{c}\begin{array}{cc}2& -2\end{array}\\ \begin{array}{cc}3& -2\end{array}\\ \begin{array}{cc}\infty & -2\end{array}\end{array}\end{array}\right)\to \left(\begin{array}{ccc}\begin{array}{c}\\ {\text{s}}_{1}\end{array}& \begin{array}{c}{\text{s}}_{1}\\ \infty \end{array}& \begin{array}{c}{\text{s}}_{2}\\ 1\end{array}\\ {\text{s}}_{2}& 0& \infty \\ \begin{array}{c}{\text{s}}_{3}\\ {\text{s}}_{4}\\ {\text{s}}_{5}\end{array}& \begin{array}{c}3\\ 3\\ 2\end{array}& \begin{array}{c}2\\ 0\\ 0\end{array}\end{array} \begin{array}{ccc}\begin{array}{c}{\text{s}}_{3}\\ 0\end{array}& \begin{array}{c}{\text{s}}_{4}\\ 2\end{array}& \begin{array}{c}\begin{array}{cc}{\text{s}}_{5}& \text{L}\end{array}\\ \begin{array}{cc}3& 0\end{array}\end{array}\\ 3& 3& \begin{array}{cc}1& 0\end{array}\\ \begin{array}{c}\infty \\ 0\\ 2\end{array}& \begin{array}{c}0\\ \infty \\ 2\end{array}& \begin{array}{c}\begin{array}{cc}0& 0\end{array}\\ \begin{array}{cc}1& 0\end{array}\\ \begin{array}{cc}0& 0\end{array}\end{array}\end{array}\right)$$

According to the meaning of the reduction number, the optimal value of the cost matrix TSP is equal to the sum between optimal value of its reduction matrix and $\text{L}$. The reduction number of cost matrixs in the travelling salesman problem is the minimum cost (objective function) of lower bound $\text{Z}(\text{m})$ in the problem root node. If $m$ becomes the node of the state space tree, $n$ is the parent node of $m$, $\overline{m }$ is the reduction matrix of $m$, the edge $<\text{x},\text{y}>$ represents an edge of the reduction matrix, the reduction value of $m$ is $\text{L}$. First, it sets all elements of the $\text{x}-\text{th}$ row and $\text{y}-\text{th}$ column of the reduction matrix of $\text{n}$ to $\infty$, which obtains the new matrix of $\text{m}$. It reduces the matrix to obtain $\overline{m }$ , so the reduced value $\text{L}$ of this step is $\overline{m }$,which gets the lower bound function Cost of non-leaf nodes, as shown in Formula (5):

5 $$\text{Z}\left(\text{m}\right)=\text{Z}\left(\text{n}\right)+\text{T}\left(\text{m},\text{n}\right)+\text{Z}\left(\overline{m }\right)$$

**Fig. 3:**
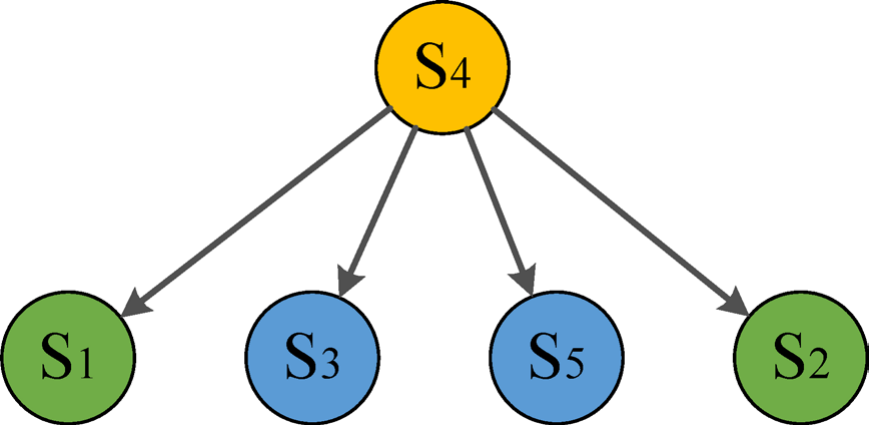
The branch and bound diagram.

**Fig. 4:**
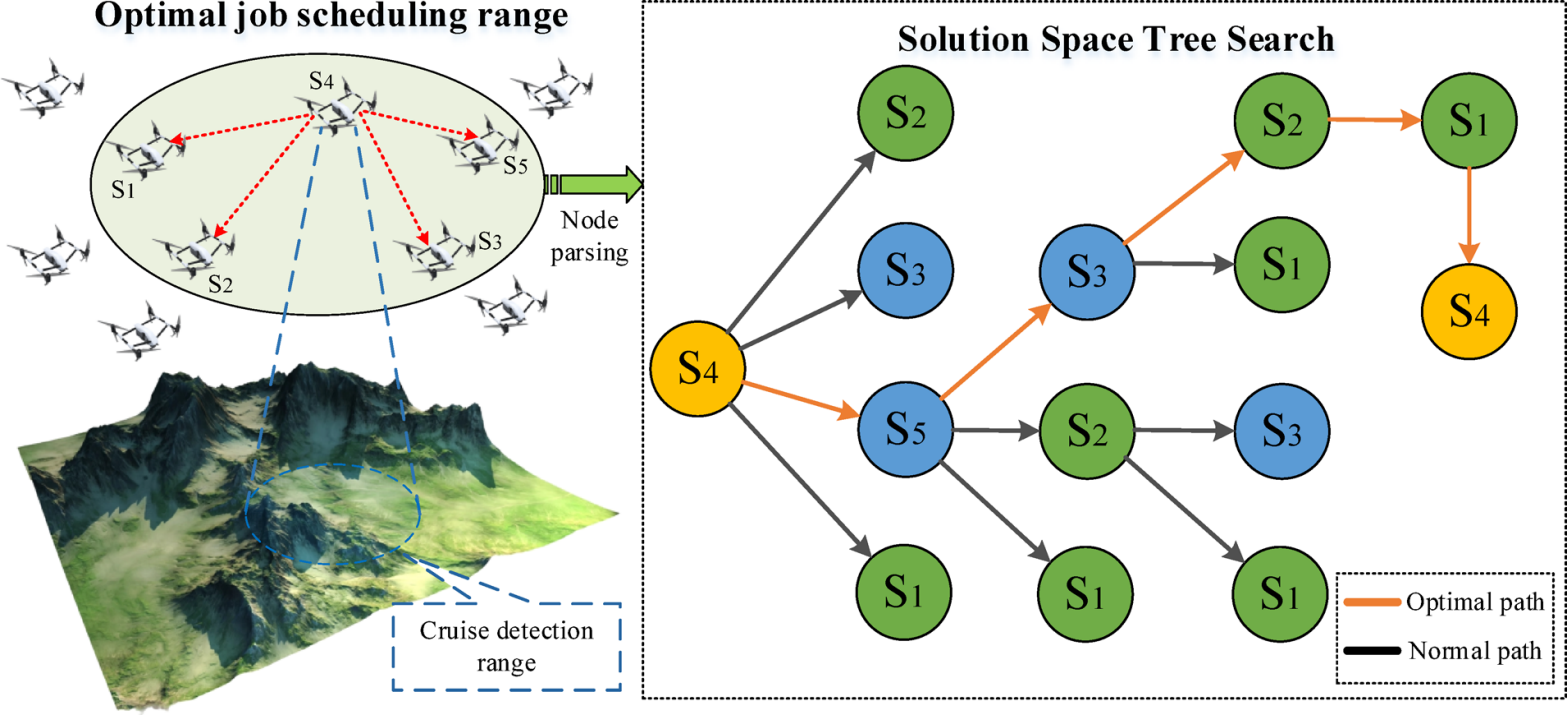
The search structure of complete solution space.

In Formula (7), $\text{Z}\left(\text{m}\right)$ represents the total cost ($\text{Z}\left(\text{n}\right)$ is the cost under the constraint), $\text{T}\left(\text{m},\text{n}\right)$ is the cost from the parent to child node, $\text{Z}\left(\overline{m }\right)$ is the cost of the current child node (the reduced value $\text{L}$). Based on the idea of the branch and bound method, the solution space tree is searched according to the cost matrix. It starts from the root node, the objective function value is calculated and added to the table of nodes, which is processed until the leaf node. Figure 3 shows the branch and bound diagram; its specific process is as follows:

1. Assume that the ${\text{s}}_{4}$ node is the starting point of job scheduling, so the root node is ${\text{s}}_{4}$, the value of the lower bound function $\text{cost}$ is ${\text{s}}_{\text{3,4}}$. It starts from ${\text{s}}_{4}$, the expandable nodes are ${\text{s}}_{1},{\text{s}}_{2},{\text{s}}_{3},{s}_{5}$,which is the bounding function value in each child node of the root node ${\text{s}}_{4}$. At the nodes ${\text{s}}_{1},{\text{s}}_{5},{\text{ and s}}_{3}$, the value of the bounding function is less than the upper bound, so the processed node can be added to the table PT. The node ${\text{s}}_{2}$ exceeds the upper bound of the objective function, so the node ${\text{s}}_{2}$ is discarded. The node with the minimum objective function value ${\text{s}}_{5}$ is selected in Table PT to prioritize the search.
2. It deals with every child node of ${\text{s}}_{2}$ in turn, which obtains the expand node ${\text{s}}_{5}$ and ${\text{s}}_{3}$. In four child nodes of ${\text{s}}_{2}$ and ${\text{s}}_{3}$, the cost of ${\text{s}}_{4}\to {\text{s}}_{5}\to {\text{s}}_{3}\to {\text{s}}_{2}$ is the smallest, so the path is ${\text{s}}_{4}\to {\text{s}}_{5}\to {\text{s}}_{3}\to {\text{s}}_{2}$. The remaining node ${s}_{1}$ returns from area A to area D. Since the node ${\text{s}}_{4}$ is a leaf node, the feasible solution is obtained, so the traversal path is ${\text{s}}_{4}\to {\text{s}}_{5}\to {\text{s}}_{3}\to {\text{s}}_{2}\to {\text{s}}_{1}\to {\text{s}}_{4}$. The complete solution space search is shown in Fig. 4.

### Improved YOLO-v8 algorithm (IM-YOLO)

This study chooses GhostNet as the lightweight optimization module of YOLO-v8, mainly based on the following three reasons:

- GhostNet generates more redundant feature maps through the Ghost module and enhances the feature expression capability at a very low computational cost, which is very suitable for drone edge computing devices.
- Compared with the traditional convolution module, the Ghost module introduces cheap operations, which can reduce the number of parameters and calculations by more than 50% and significantly improve the inference speed while maintaining the detection accuracy.
- In agricultural scenes, especially in complex backgrounds, targets often present small size, blurred texture and other characteristics. GhostNet can more effectively enhance detail features and help improve the recognition rate of small targets.

**Fig. 5:**
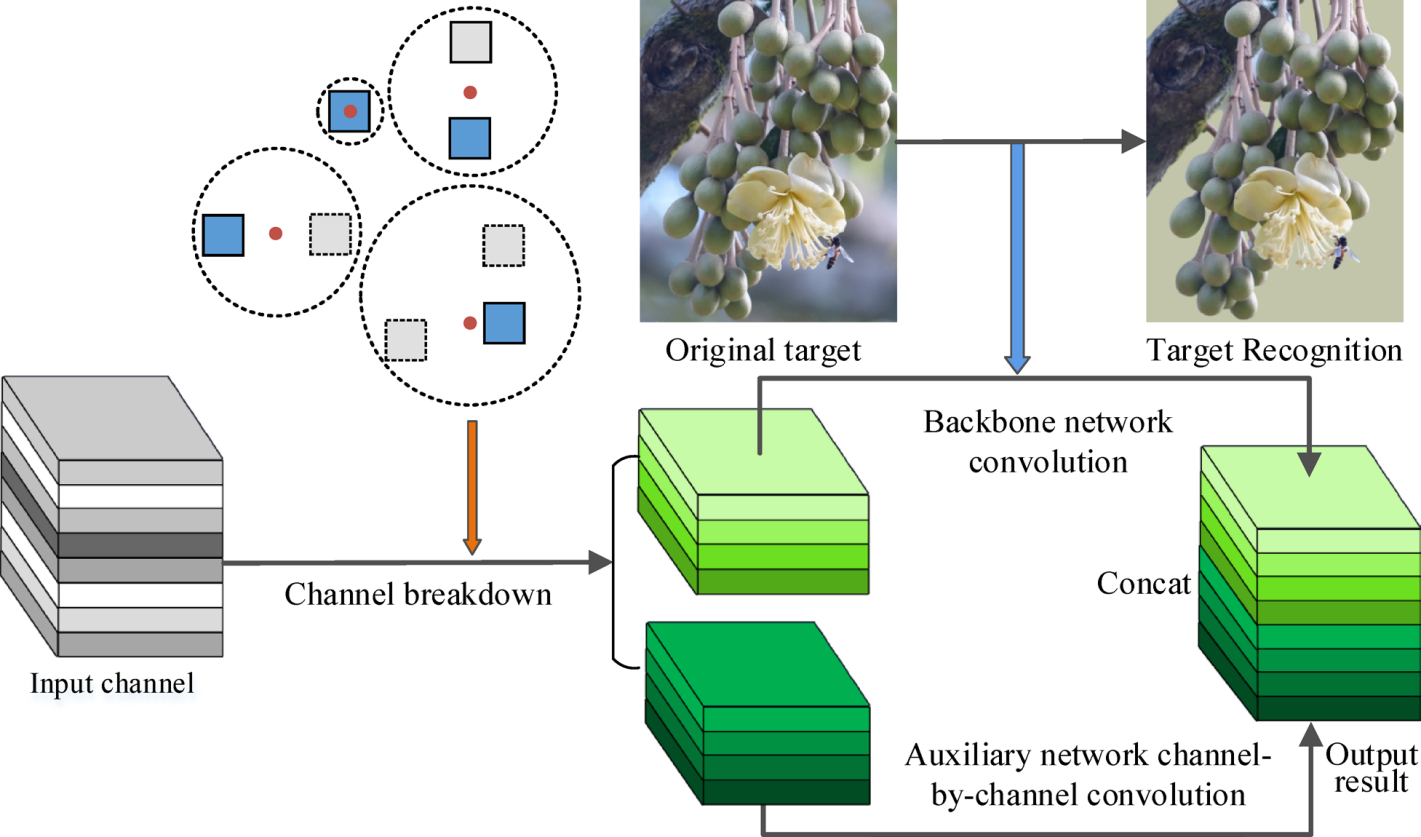
The operation process of Ghost Module channel convolution.

Therefore, GhostNet is a cost-effective deep learning feature extraction structure suitable for deployment on drone platforms. The branch and bound method is the most suitable basic optimization framework for path planning in this study. When the drone completes route planning and navigates to the target area, it will begin to identify the target object and carry out durian pollination operations. The YOLO-v8 (You Only Look Once version 8.0) is an efficient target detection algorithm suitable for the real-time target detection^24^. However, when the YOLO-v8 algorithm extracts feature maps, there will be a large number of redundant and similar feature maps. These feature maps are indispensable for the algorithm’s accuracy, which are obtained by convolution operations and input into the next convolutional layer for operation. It involves a large number of network parameters and consumes many computing resources. In order to use lower-cost computing to obtain these redundant feature maps, this study uses the GhostNet lightweight network to optimize the YOLO-v8 algorithm^25^. It uses a new module called Ghost Module to improve the performance of the algorithm. The Ghost Module divides the input channel into two parts, one for the convolution operation of the backbone network and the other for the sub-channels that are used to assist the network in the channel-by-channel convolution operations, which reduces the number of convolutions kernels and uses the squeeze-and-excitation module to enhance the feature representation capability. Figure 5 shows the operation process of Ghost Module channel convolution. The main steps are follows:

- First, half of the convolution kernels are used to perform the traditional convolution operations.
- Use the other half of the 3 × 3 convolution kernel to perform the channel-by-channel convolution operations, which is called the cheap operations.
- Concat the outputs of two parts.

In the FLOPs (Floating Point Operations Per Second) calculation of the conventional convolution,$\text{a}$ is the height of the output, $\text{b}$ is the width of the output,$\text{c}$ is the output dimension (the number of convolution kernels), $\text{e}$ is the number of channels, and $\text{r}$ is the height and width of the convolution kernel. This study uses the Ghost module to improve it, which divides $\text{c}$ groups convolution kernel of the conventional convolution operation into $\text{k}$ groups. $\upvarphi$ is the convolution kernel of the Cheap operations, which is approximately equal to $\text{r}$, so the speedup ratio $\text{f}(\text{k})$ and compression ratio $\text{f}(\text{e})$ are shown in Formula (6 and 7). The convolution kernel is divided into two groups, so the value of $\text{k}$ is 2. The number of parameters and calculation time of the Ghost module is one-half of the conventional convolution.

6 $$\text{f}\left(\text{k}\right)=\frac{\text{e}\times \text{r}\times \text{r}}{\frac{\text{c}}{\text{k}}\times \text{e}\times \text{r}\times \text{r}+\frac{(\text{k}-1)}{\text{k}}\times \upvarphi \times \upvarphi }\approx \frac{k\times \text{r}}{k+\text{r}-1}\approx \text{k}$$

7 $$\text{f}\left(\text{e}\right)=\frac{\text{c}\times \text{e}\times \text{r}\times \text{r}}{\frac{\text{c}}{\text{k}}\times \text{e}\times \text{r}\times \text{r}+\frac{(\text{k}-1)\text{c}}{\text{k}}\times \upvarphi \times \upvarphi }\approx \frac{k\times \text{e}}{k+\text{e}-1}\approx \text{k}$$

**Fig. 6:**
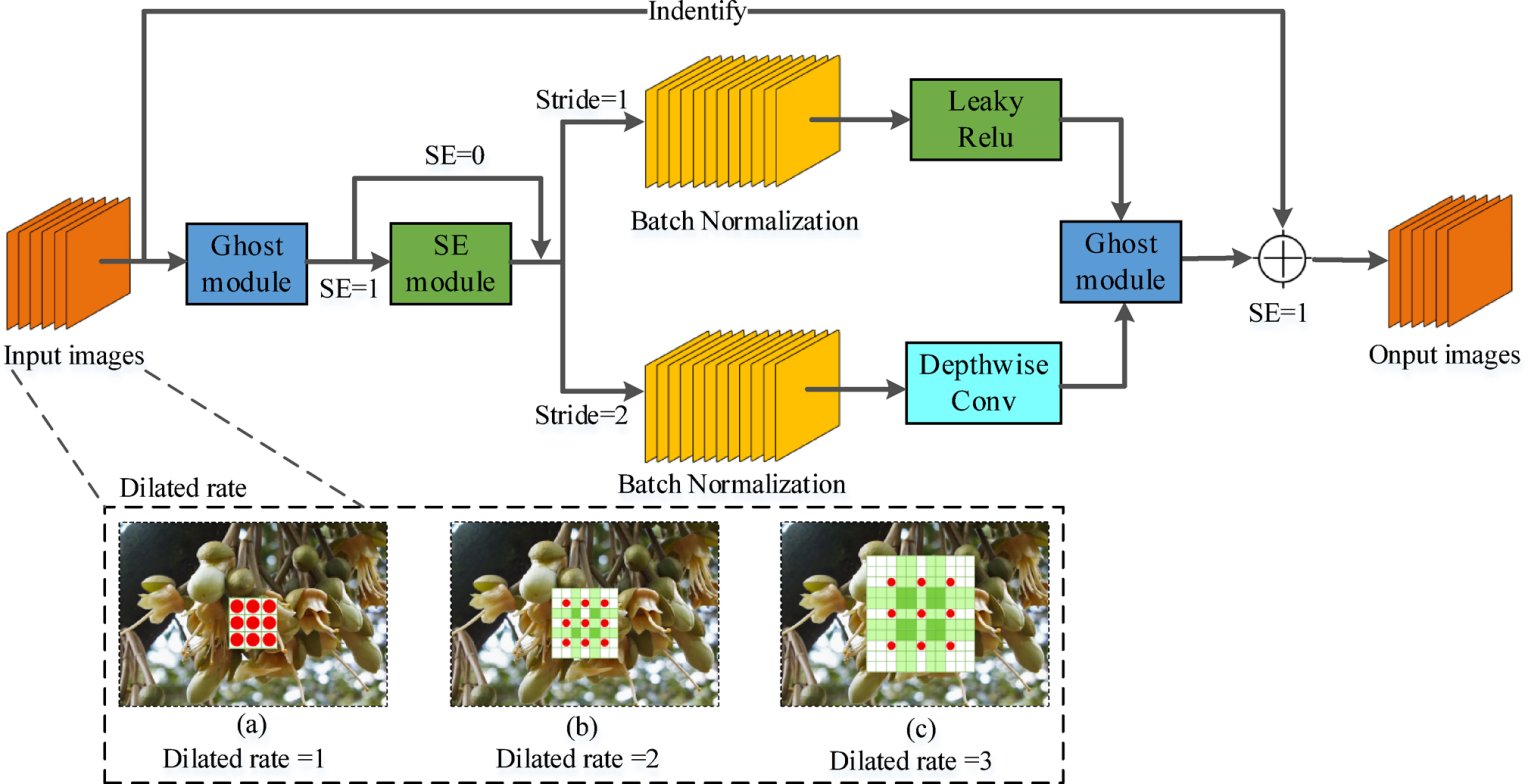
The structure of Ghost-BottleNeck module.

Regarding the lightweight characteristics of the Ghost module, this study proposes the Ghost-BottleNeck module^26^, which is shown in Fig. 6. The module consists of two stacked Ghost modules and the BN layer is added to accelerate the convergence of the network and prevent the overfitting. The first part introduces the Leaky Relu activation function to prevent the problem of not-learning neurons in the process. The second part does not introduce the activation function to keep the data in the same distribution during training, which can speed up the convergence of the model.

**Fig. 7:**
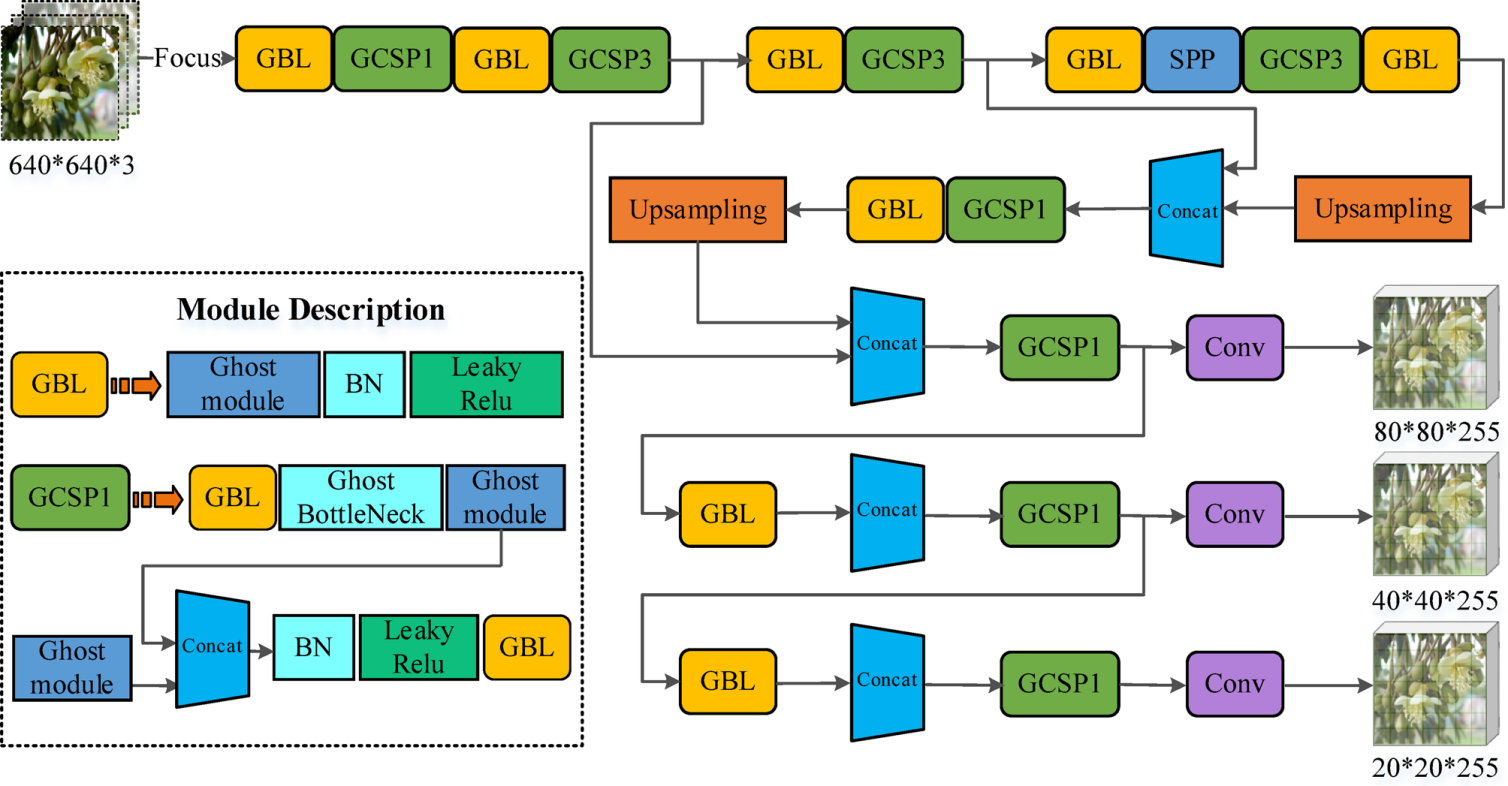
The overall structure of IM-YOLO network.

Based on the Ghost module and Ghost-BottleNeck module, this study improves the CBL module and CSP_X in the YOLO-v8 network structure, which obtains the GBL and GCSP_X. Compared with the original YOLO-v8, the YOLO-v8 Ghost model only uses the CSP_X structure, which reduces the complexity of the model. It completely transfers the information of gradient changes to the feature map, optimizes the feature fusion ability of the network, and thus ensures the accuracy of detection. Figure 7 shows the overall structure of IM-YOLO network.

In this study, GhostNet was introduced into the backbone feature extraction network of YOLO-v8 to replace some standard convolution modules and CSPBlock, thereby achieving lightweight optimization. GhostNet mainly generates redundant feature maps through the Ghost Module structure and uses cheap operations (such as point-by-point convolution + depth-separable convolution) instead of standard convolution operations, fundamentally reducing the number of parameters and calculations. In the model implementation, this study retains the basic framework of the YOLO-v8 backbone network, focusing on replacing the convolution structure of the 3rd to 5th layers with the Ghost-Bottleneck structure, retaining the residual connection, and enhancing the expression of low-level semantic information. In order to quantify the optimization effect after the introduction of GhostNet, this study set up a comparative experiment with the original YOLO-v8. Table 1 shows the YOLO-v8 performance results before and after the introduction of the GhostNet module. It shows that IM-YOLO reduces the model size by about 37.6%, shortens the inference time by 36%, increases the detection frame rate by about 52%, and significantly improves the detection accuracy by about 11.6%. This optimization result shows that GhostNet reduces the model size and improves the real-time detection capabilities in complex backgrounds and low-light scenes and is a key basic module for implementing edge computing deployment. In addition, in the model deployment test, the frame rate of IM-YOLO running on Jetson Xavier NX is stable at 28 ~ 32 fps, indicating that it has good real-time and portability on embedded devices, and is suitable for the field deployment needs of drone pollination systems.

**Table 1:** The YOLO-v8 performance results before and after the introduction of the GhostNet module.

Model	Model size (Params)	FLOPs (GFLOPs)	Inference time (ms/img)	Detection speed (FPS)	mAP@0.5
YOLO-v8 (original)	9.3 M	14.7	19.2	52.1	79.86%
IM-YOLO (with GhostNet)	5.8 M	8.5	12.3	79.3	91.43%

## Experimental results

In order to test the performance of the EN-TSP and IM-YOLO algorithm, this study compares the EN-TSP algorithm with the TSP, ACO (Ant Colony Optimization)^27^ and PSO (Particle Swarm Optimization)^28^ path planning algorithm, it compares the IM-YOLO algorithm with the YOLO-v8, Faster RCNN(Region-based Convolutional Neural Network)^29^ and EfficientDet^30^ detection algorithms. The experimental results are as follows:

### Navigation effect

**Fig. 8:**
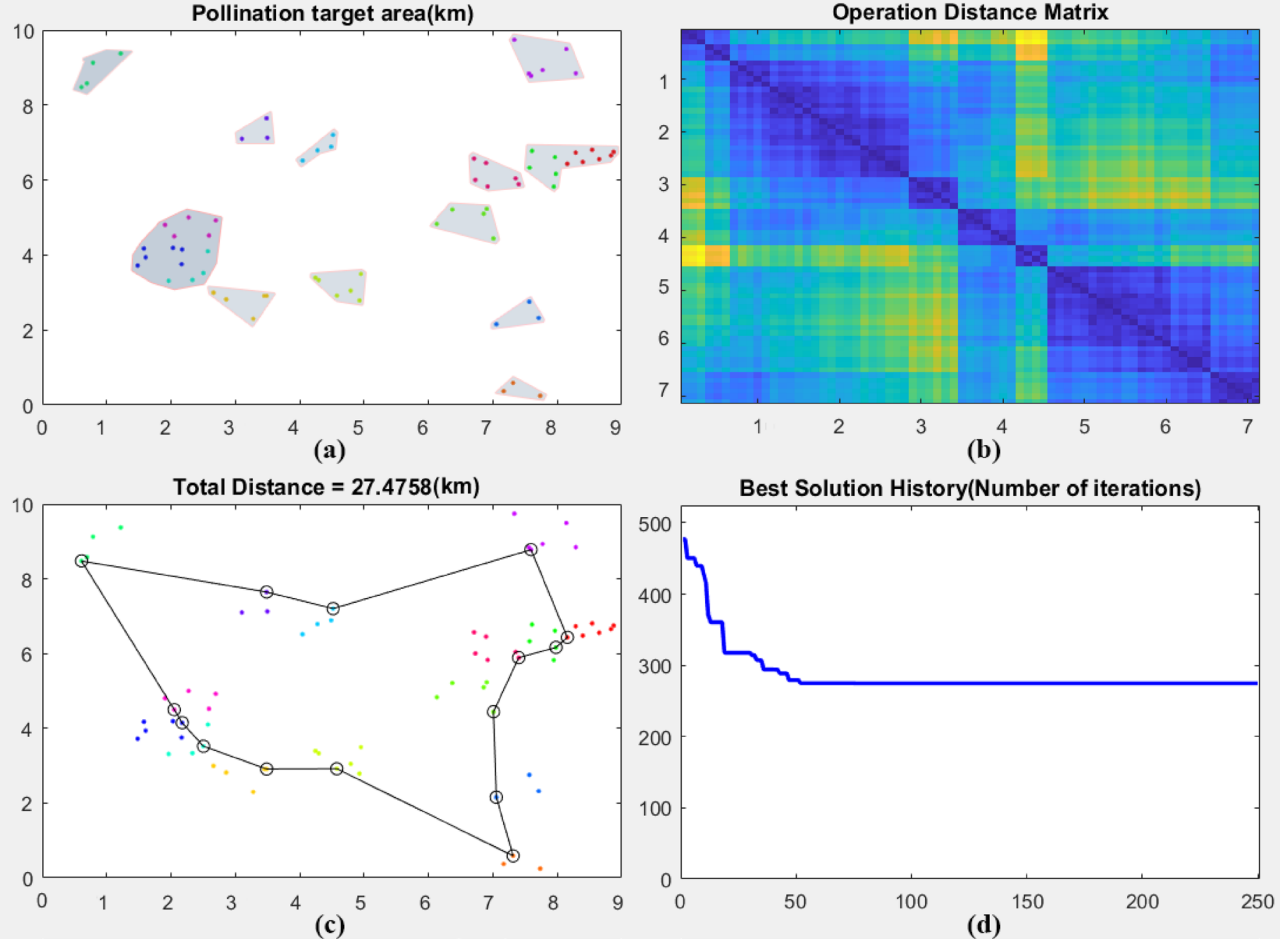
The shortest trajectory and iteration of the EN-TSP algorithm to control the drones’ pollination operation.

Figure 8 shows the shortest trajectory and iteration of the EN-TSP algorithm to control the drones’ pollination operation. The irregular squares represent the pollination area, the points of the area represent the pollination targets. The EN-TSP algorithm uses the distance matrix between the operation areas and evaluates the distance relationship between the areas according to the distribution of the target areas for the drone to perform pollination tasks, which determines the optimal route. The simulated flight range of the drone is 27.4758 km. The drone starts from the starting coordinates (0.75, 8.5), then it passes through multiple pollination operation areas, which are optimized according to the distribution of durian flowers and the shortest distance of the drone’s navigation path. The order of the operation areas follows the optimal solution of the EN-TSP algorithm. After completing all pollination tasks, the drone returns to the starting point according to the planned route. Finally, in order to ensure that the route is the optimal value, the EN-TSP algorithm performs multiple iterations and stabilizes after 50 times, and the final route gradually tends to be the optimal value. Because the EN-TSP algorithm optimizes the TSP scheduling algorithm through the branch and bound method, which finds the globally optimal path in complex path planning problems, avoids the risk of falling into the local optimal solution and improves the overall operation efficiency.

In order to verify the scheduling performance of the EN-TSP algorithm, this study introduces three scheduling algorithms (TSP, ACO and PSO) for simulation tests. It uses the TPE (total range error), RMSE (Global Root Mean Square Error) and TC (Trajectory Coverage) to evaluate the global consistency of the entire trajectory. It also uses LPE (Local Position Error) and LRMSE (Local Path Root Mean Square Error) to evaluate the accuracy of the local trajectory. Table 2 shows the performance comparison results of path planning algorithms. Compared with the TSP, ACO and PSO algorithm, the TPE value of EN-TSP algorithm is reduced by 2.29, 3.25 and 5.63 m; the RMSE value is reduced by 2.19, 1.72 and 1.17 m; the TC value is increased by 12.89%, 9.29% and 4.51%; the LPE value is reduced by 0.89, 0.46 and 0.23 m; the LRMSE value is reduced by 1.34, 0.79 and 0.37 m. This is because the EN-TSP algorithm optimizes the TSP scheduling algorithm through the branch and bound method, which find the global optimal path more effectively when dealing with complex path planning problems. It also avoids the problem of falling into the local optimal solution and ensures that the drone can complete the task with a shorter total range (TPE). The local performance is also superior, the drone controlled by the EN-TSP algorithm can fly more accurately in each small area according to the predetermined path, which reduces the risk of deviating from the predetermined trajectory.

**Table 2:** The performance comparison results of path planning algorithms.

Algorithm	TPE(m)	RMSE(m)	TC(%)	LPE(m)	LRMSE(m)
TSP	6.85	2.76	83.43	1.25	1.89
ACO	4.47	2.29	87.03	0.82	1.34
PSO	3.51	1.74	91.81	0.59	0.92
EN-TSP	1.22	0.57	96.32	0.36	0.55

### Target detection simulation effect

**Fig. 9:**
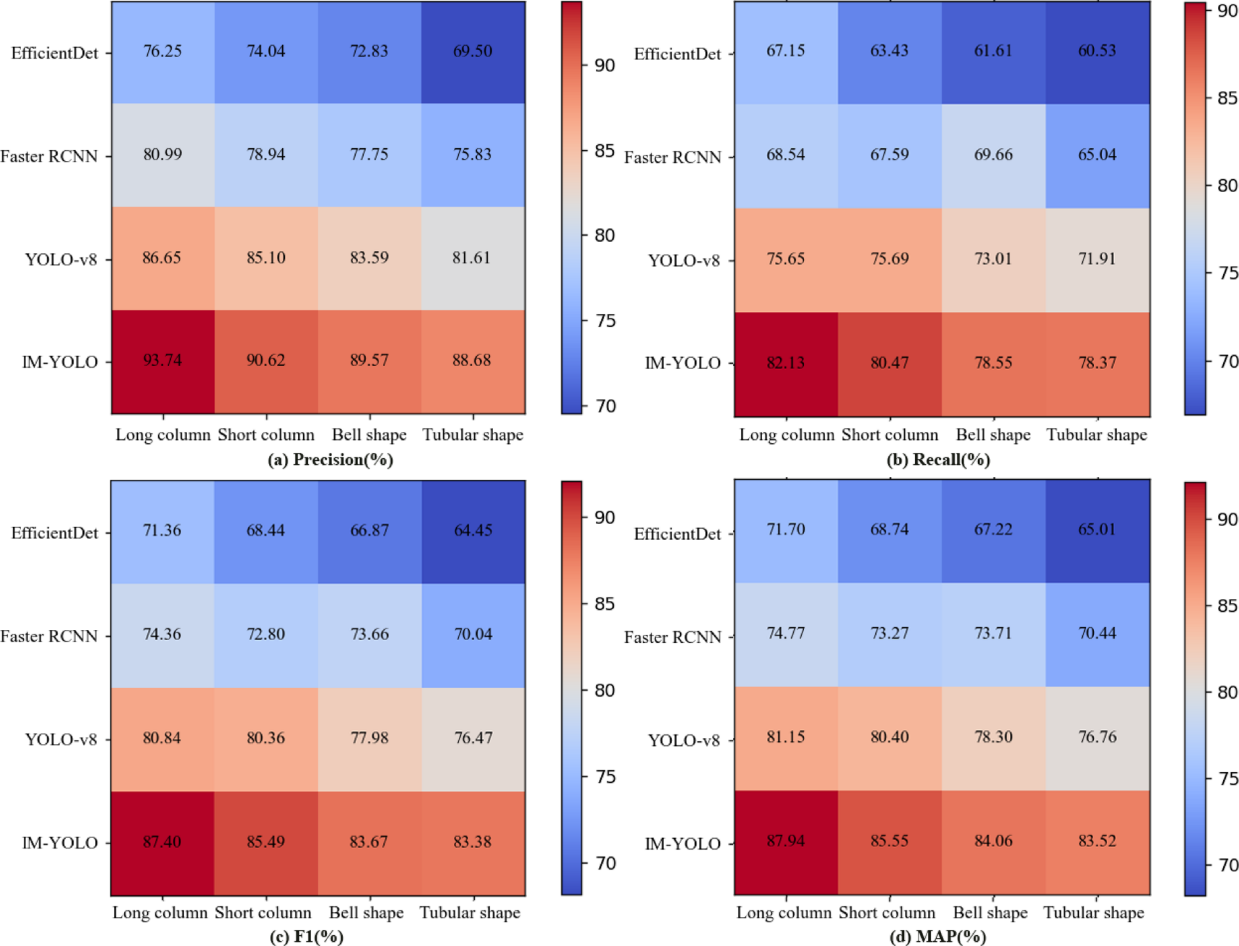
The confusion matrix analysis results of four algorithms identifying four forms of durian flowers.

In order to test the recognition performance of the IM-YOLO algorithm, this study uses four types of images of durian flowers (long columns, short columns, bell and tube) as experimental objects. Table 3 shows the detection effects of each target detection algorithm on the four types of durian flowers. The Precision, Recall, F1 and MAP values of the IM-YOLO algorithm are 90.68%, 79.92%, 85.28% and 85.84%. Compared with the EfficientDet, Faster RCNN and YOLO-v8 algorithm, it improves 25.40%, 15.02% and 5.85% in Precision; 23.28%, 18.22% and 6.73% in Recall; 26.10%, 15.90% and 7.01% in F1; 25.88%, 17.46% and 6.53% in MAP. Figure 9 shows the confusion matrix analysis results of four algorithms in identifying four kinds of durian flowers. It shows that the performance of the IM-YOLO algorithm in identifying four kinds of durian flowers (long column, short column, bell and tube) is better than other three algorithms. The above results show that the detection accuracy of the IM-YOLO algorithm is higher than the YOLO-v8, Faster RCNN and EfficientDet algorithms in the overall or single category. Because GhostNet uses the strategy based on deep separable convolution to generate more feature maps and uses fewer parameters to achieve more feature representations of the YOLO-v8 algorithm. It also optimizes the feature fusion, which allows the YOLO-v8 algorithm to fuse features of different scales more efficiently when detecting them, so it improves the overall algorithm performance.

**Table 3:** The detection effects of each target detection algorithm on the four types of durian flowers.

Model	Precision (%)	Recall (%)	F1 (%)	MAP (mean average precision) (%)
EfficientDet	72.31	64.83	67.63	68.19
Faster RCNN	78.84	67.60	73.58	73.08
YOLO-v8	85.67	74.88	79.69	80.58
IM-YOLO	90.68	79.92	85.28	85.84

### Ablation analysis

In order to analyze the role of "minimum cost pruning strategy" and "state space reduction method" in the EN-TSP algorithm, this study designed a module ablation experiment. Table 4 shows the ablation experiment results of different module combinations of EN-TSP. After the introduction of the minimum cost pruning strategy, the path calculation time is reduced from 13.5 s to 10.1 s, and the average path length is shortened by 7.2%, indicating that the cost lower bound strategy can effectively optimize the search order. Further adding the state space reduction mechanism, the calculation time is shortened to 8.6 s, the path planning accuracy is further improved by 11.8%, and the memory usage is reduced by about 23.6%. It shows that EN-TSP effectively solves the computational redundancy problem of traditional TSP in multi-region pollination tasks while maintaining the optimality of the path. Overall, the EN-TSP module significantly improves the operation efficiency of the algorithm while ensuring the path quality by optimizing the search space and cost estimation mechanism, which is especially suitable for dynamic and complex orchard path planning tasks.

**Table 4:** The ablation experiment results of different module combinations of EN-TSP.

Module	Pruning strategy	Cost matrix reduction	Avg. path length (m)	Avg. computation time (s)	Peak memory usage (MB)	Path accuracy improvement (%)
Original TSP	No	No	147.8	13.5	235.4	–
TSP + Pruning	Heuristic	No	137.2	10.1	210.7	↑ 7.2%
Full EN-TSP	Heuristic	Yes	130.3	8.6	179.8	↑ 11.8%

In order to verify the impact of ablation experiments on the various durian flower detection, this study divides the ablation experiments into YOLO-v8, YOLO-v8 + MobileNetv3^31^, YOLO-v8 + ECA (Efficient Channel Attention)^32^, YOLO-v8 + Ghost-BottleNeck. Table 5 shows the ablation experiment results of different module combinations of IM-YOLO. Compared with the YOLO-v8 algorithm, the memory usage of the YOLO-v8 + MobileNetv3 model is reduced by 19.62%, but the MAP, F1 and FPS are only reduced by 3.77%, 3.72% and 5.92%. Because the greatly simplified network structure of MobileNetv3 weakens the recognition channel mechanism. The MAP, F1 and FPS of the YOLO-v8 + ECA model are improved by 12.71%, 10.34% and 19.47%, but the memory usage is only reduced by 3.59%. Because the appropriate cross-channel interaction strategy adopted by ECA can maintain the detection performance but increase the model complexity. The memory usage of YOLO-v8 + GhostNet is reduced by 23.68%, and the MAP, F1 and FPS are improved by 13.94%, 11.39% and 20.59%. It shows that the IM-YOLO can fuse features of different depths to enhance the expression of shallow semantic information and use the effective channel attention to enhance the feature information of important channels. It can capture more feature information while maintaining low computational complexity, which enables the network to extract richer features without increasing the computational burden, so it can improve the detection accuracy.

**Table 5:** The ablation experiment results of different module combinations of IM-YOLO.

Module	Memory usage (%)	MAP (%)	F1 (%)	FPS (frames/s)
YOLO-v8	87.17	80.15	79.61	65.75
YOLO-v8 + MobileNetv3	72.87	83.17	82.57	69.64
YOLO-v8 + ECA	84.15	90.34	87.84	78.55
YOLO-v8 + Ghost-BottleNeck	70.48	91.32	88.68	79.29

### Resource analysis

In order to comprehensively evaluate the application potential of path planning algorithms in actual UAV scheduling tasks, this study also compared the running time and computing resource consumption of the four algorithms EN-TSP, TSP, ACO and PSO. The experiment was conducted on the same hardware platform (Intel Core i9-10900 K + 64 GB RAM), and the average solution time of each algorithm in completing 17 regional path planning tasks was measured. The results show that the traditional TSP is faster in small-scale regional planning, but after the number of nodes exceeds 15, the path search time increases exponentially, and the average running time reaches 13.5 s. ACO and PSO are faster in the early stage of convergence, but there is oscillation in the later iteration process, resulting in an overall time of 16.7 s and 14.9 s respectively. EN-TSP uses the minimum cost branch and bound method and greatly compresses the search space through pruning strategies. It significantly improves the computational efficiency while ensuring global optimality. The final average solution time is only 8.6 s, which is 48.5% faster than ACO and 42.2% faster than PSO. In terms of memory usage, EN-TSP uses the cost reduction matrix to quickly determine the lower bound, avoiding unnecessary path expansion. The peak memory usage is about 180 MB, which is lower than ACO (245 MB) and PSO (213 MB). At the same time, EN-TSP’s path selection logic is more compact, avoiding the additional redundant calculations caused by pheromone updates or particle position updates in traditional heuristic algorithms. Overall, EN-TSP has both lower time complexity and less resource usage, and is an efficient path optimization algorithm suitable for multi-objective path planning in complex orchards.

In addition to the improvement in detection accuracy, IM-YOLO also shows significant advantages in runtime performance and resource usage. The experiment compared the performance of YOLO-v8, Faster RCNN, EfficientDet and the improved IM-YOLO model in terms of running speed (FPS) and memory usage. The results show that IM-YOLO has a frame rate of 79.29 fps on the NVIDIA RTX 3080 platform, which is significantly higher than YOLO-v8 (65.75 fps), EfficientDet (62.31 fps) and Faster RCNN (58.64 fps), indicating that it has stronger processing capabilities in real-time applications. In terms of memory usage, IM-YOLO uses GhostNet and Ghost-BottleNeck modules, which greatly reduces the calculation of redundant feature maps. The overall model parameter volume is controlled at 5.8 M, and the floating-point operation complexity is only 8.5 GFLOPs. The ablation experiments further verified that YOLO-v8 + Ghost-BottleNeck reduced memory usage by 23.68% compared to the original YOLO-v8, while MAP increased by 13.94% and F1 score increased by 11.39%. In contrast, although MobileNetv3 also reduced memory usage, it suffered a certain loss in accuracy indicators; while the ECA model improved accuracy significantly, but the memory savings were not significant. In summary, IM-YOLO effectively controls resource consumption while taking into account both detection accuracy and computational efficiency, and is feasible for deployment on resource-constrained drone platforms, reflecting good engineering practicality and application potential promotion.

### Simulation test

**Fig. 10:**
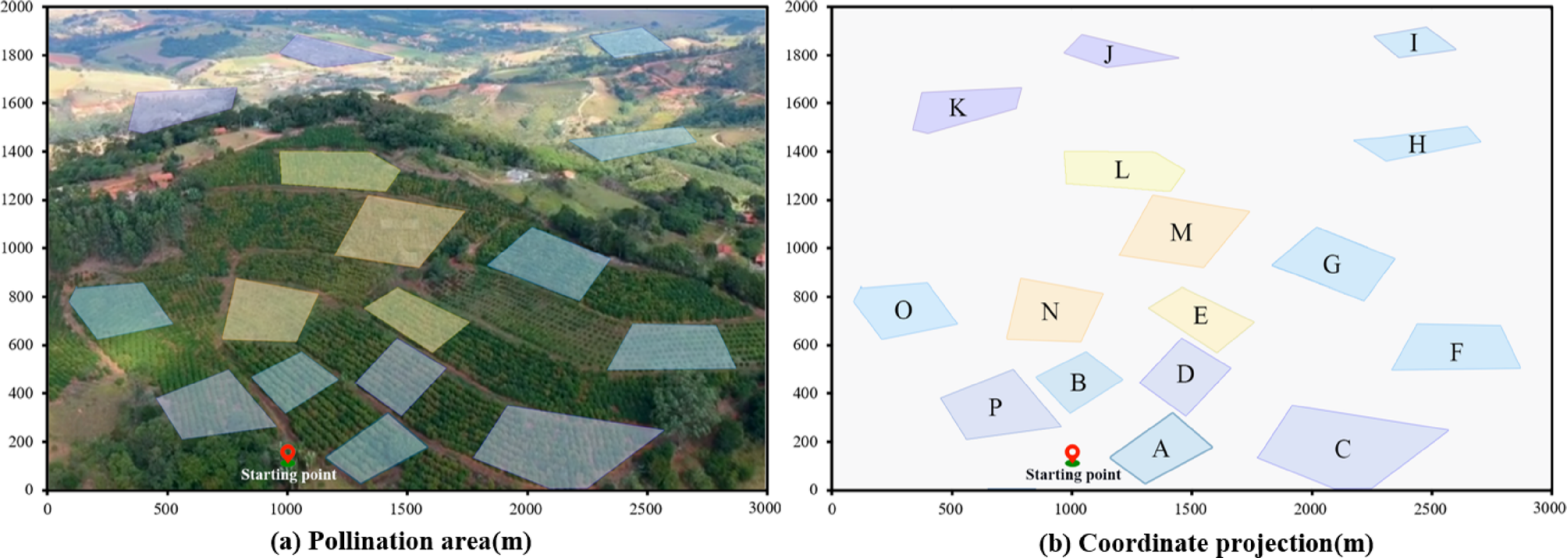
The stereoscopic projection of the durian pollination experimental area.

In order to further verify the performance of the EN-TSP and IM-YOLO algorithms, the drone first uses the route planning algorithm to calculate the optimal path from the starting point to all nodes and back to the endpoint in multiple pollination areas. After entering each area, the target recognition algorithm is used to detect the durian flowers in the area and perform operations. This study also uses a single-area modelling method to perform the coordinate projection on the above 12 durian flower pollination areas, as shown in Fig. 10. The thick blue solid lines are the boundaries of each area, the light-colored areas are each pollination operation area, and the red solid points on the boundaries are the entry and exit points.

**Fig. 11:**
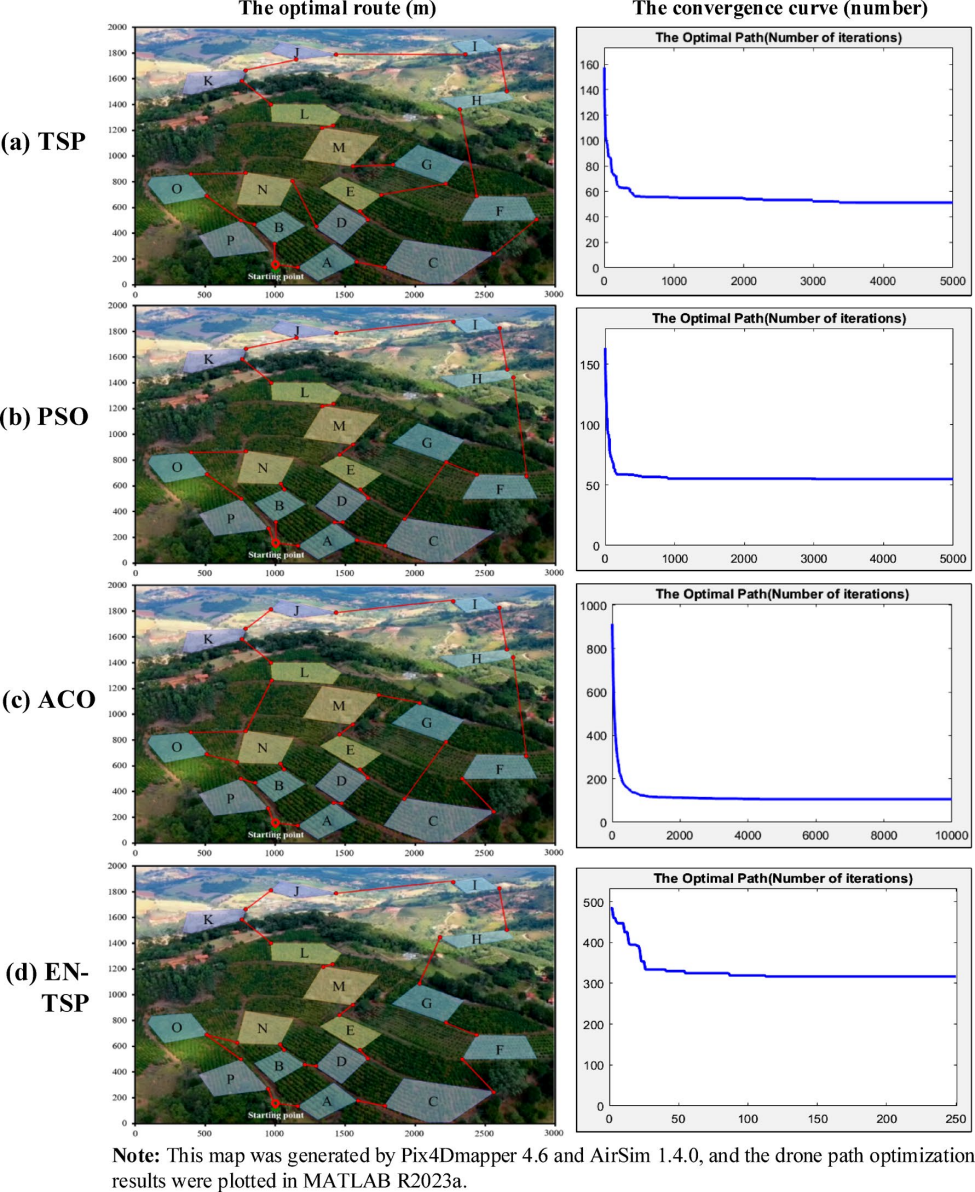
The drone route planning and curve optimization of four algorithms.

Figure 11 shows the drone route planning and curve optimization of four algorithms (The map shown in this figure uses Pix4D software to reconstruct the two-dimensional image taken by the drone into a three-dimensional orchard model, which is then imported into the Microsoft AirSim simulation platform (v1.4.0) to build a drone pollination path simulation environment. The path visualization and optimization results are plotted and processed using MATLAB R2023a). The route sequence of the TSP algorithm is A → C → F → H → I → J → K → L → M → G → E → D → N → O → P → B → A. Since the TSP algorithm only considers the simple path-planning problems, it falls into the optimal local solution when dealing with larger-scale problems. The drone has obvious detours in some areas, which increases the overall flight distance. Its convergence speed is slow, and the initial path adjustment amplitude is large, indicating that the algorithm adjusts the path during the iteration process, but the final result is not ideal. The route sequence of the PSO algorithm is A → C → G → F → H → I → J → K → L → M → E → D → A → B → N → O → P → A. Although it is optimized relative to the TSP algorithm, it is prone to fall into local cycles, which causes repeated flights in certain areas. This repetition increases the flight distance and time, which cannot find the global optimal solution well. Its convergence is fast, but the path optimization effect is weakened in the middle of the iteration, and it finally converges to the suboptimal solution. The route sequence of the ACO algorithm is A → D → E → M → G → C → F → H → I → J → K → L → N → O → N → B → P → A. The ACO algorithm simulates ant colony behaviour to search for paths. The early convergence speed is slow, and it takes multiple iterations to find the better solution. The path planning of the drone in some areas still has unnecessary repetitions and detours, which increases the hovering time and reduces efficiency. Its convergence speed is moderate, but it requires more iterations to converge to the better path. The route sequence of the EN-TSP algorithm is A → C → F → G → H → I → J → K → L → M → E → D → B → N → O → P → A. The drone starts from the starting point and completes the pollination tasks of each area in turn according to the optimal path. Compared with other three algorithms, the EN-TSP algorithm effectively avoids the problem of locally optimal solutions, which ensures that the drone can cover all pollination areas with the shortest path and return to the starting point according to the original path. Its convergence speed is fast, and the path tends to be stable in the early iterations, which shows the excellent global optimal path search capabilities. The navigation distances of TSP, ACO, PSO and EN-TSP algorithms are 9.66 km, 7.38 km, 6.89 km and 5.43 km. Compared with other three algorithms, the navigation distance of EN-TSP is shortened by 77.90%, 35.91% and 26.89%. It shows that the EN-TSP algorithm can guarantee the global optimal solution and reduce the computational complexity so that it performs better in the route planning of multi-objective tasks, which can effectively avoid local optimal and dead loop problems to ensure the optimal path.

**Fig. 12:**
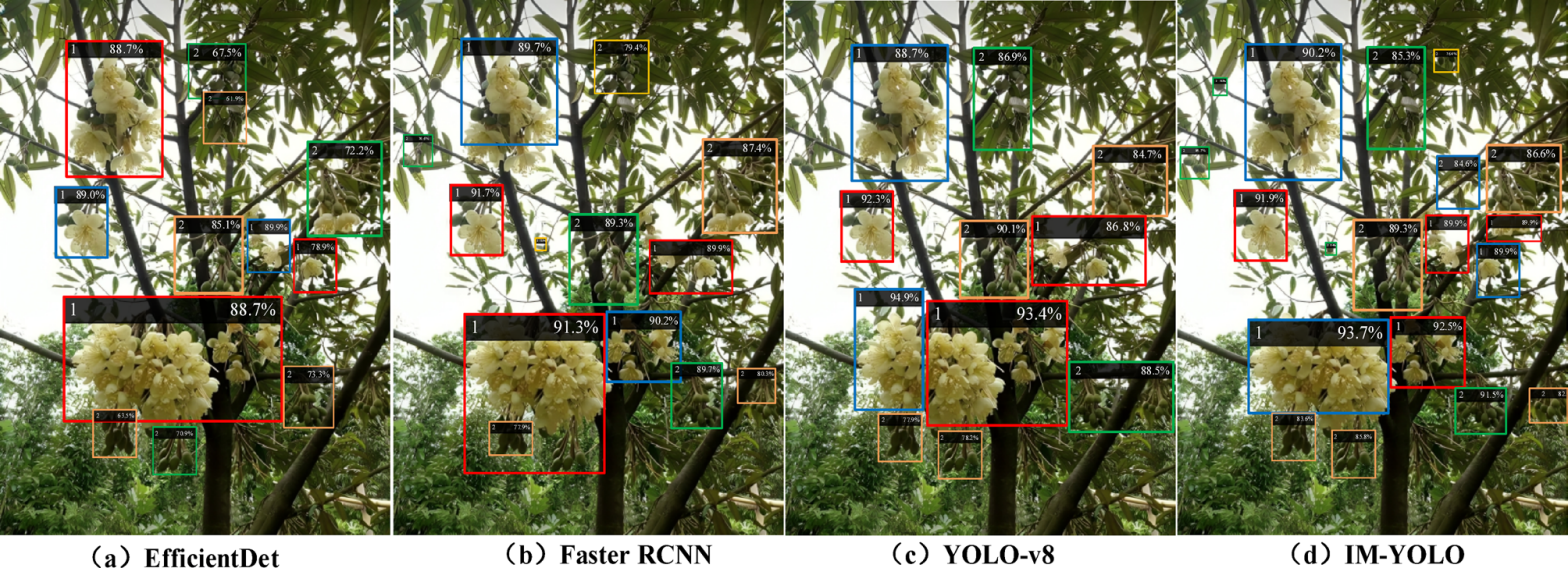
The target detection effect of durian flowers (1: Durian flower, 2: Durian flower bud).

After the drone determines the route for the pollination operation of the durian flower, the next step is to identify the pollination targets in each small area and carry out the pollination operations. In order to demonstrate the recognition effect of each algorithm, the target detection effect of durian flowers is shown in Fig. 12. The results show that the IM-YOLO algorithm performs well and can complete different recognition and positioning. The other three algorithms have some shortcomings. The EfficientDet algorithm is difficulty to recognizes bell-shaped durian flowers in feature extraction, when the lighting conditions are poor and other backgrounds are complex. The Faster RCNN algorithm is not accurate in locating the durian flower target, and its two-stage detection method reduces the detection accuracy under low light conditions. The YOLO-v8 algorithm is limited in its feature extraction and fusion capabilities for relatively small and unclear durian flower targets, and the recognition accuracy is reduced. The IM-YOLO algorithm is improved by the GhostNet module, it generates more virtual feature maps, so it can better capture the details of the durian flower under low light conditions and improve the feature extraction ability. The IM-YOLO algorithm reduces the demand for computing resources and achieves efficient feature extraction, which can capture the morphological differences of durian flowers and improve the detection accuracy of small targets.

**Fig. 13:**
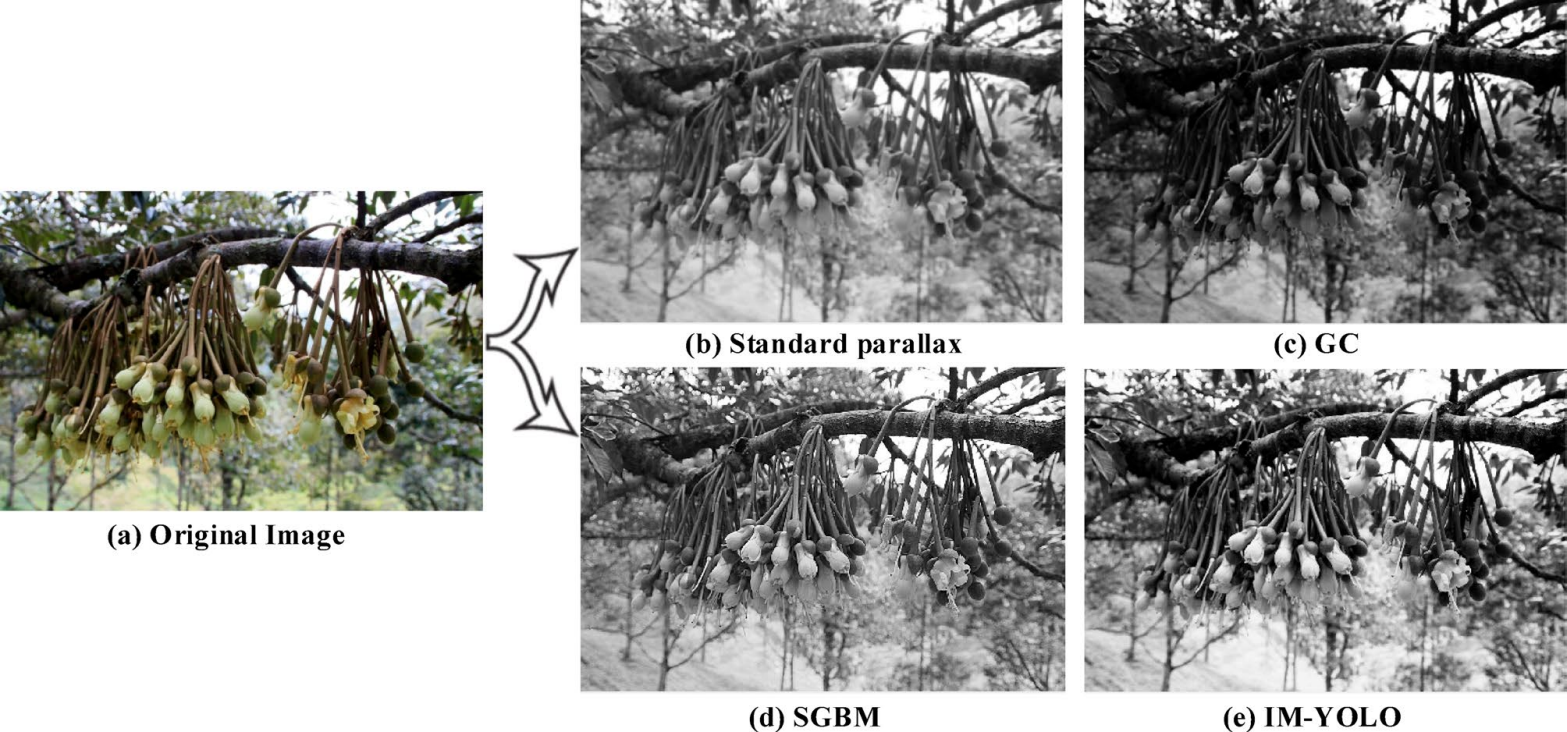
The dense disparity maps of different algorithms.

Figure 13 shows the dense disparity maps of different algorithms. The standard disparity algorithm provides the basic depth difference information on durian flowers and capture the depth changes of durian flowers at different distances; however, it performs poorly in processing details and generates noise in the occluded or texture less areas. Compared with the standard disparity algorithm, the GC (graph cut) algorithm uses the global optimization method to estimate disparity and improve performance. The graph cut method minimizes an energy function that includes smoothness and data consistency, which generates the smooth disparity map of durian flowers. However, it has high computational complexity and performs poorly in the texture less areas or repetitive pattern areas. The SGBM (semi-global block matching) algorithm^33^ combines the local block matching and semi-global optimization to strike the balance between accuracy and global consistency, which performs well in areas with fine textures and small disparity changes. However, it introduces the artefacts in low-contrast areas of this image and is sensitive to noise. The IM-YOLO algorithm uses the GhostNet module to generate high-precision and robust disparity maps. It captures more details of the durian flower, especially in the durian garden environment with large texture changes. It also reduces computational complexity and improves the detection accuracy.

### Results analysis

This study solves the problems of complex target detection and efficient path planning in durian pollination scenarios by proposing the IM-YOLO and EN-TSP algorithms. Compared with existing target detection algorithms, IM-YOLO outperforms Faster RCNN, EfficientDet and other methods in detection accuracy, recall rate and processing efficiency, especially showing stronger robustness under complex backgrounds and low light conditions. Compared with existing path optimization algorithms, EN-TSP achieves global optimal path planning through the branch and bound method, which is significantly better than TSP, PSO and ACO algorithms, not only improving the efficiency of path optimization, but also reducing the energy consumption of the task. The experimental results further verified the practical applicability and innovation of the proposed method and provided a solid theoretical basis and technical support for the application of agricultural UAVs in complex scenarios.

Although the results of this study are very convincing, the future research will also enhance the depth and breadth of the discussion from error analysis, ablation analysis and benchmark testing. The first is error analysis. Since the color and texture of durian flowers and the background are highly similar, the IM-YOLO algorithm is prone to false detection or missed detection in areas covered by high-density vegetation. In low-resolution input images, there is insufficient target detail information, resulting in reduced feature expression capabilities and reduced recognition accuracy. Under low light or backlight conditions, the contrast of the target area is low, making it difficult for the IM-YOLO algorithm to accurately capture the target edge information. In response to the above problems, in the future, algorithm performance can be improved by enhancing the diversity of data sets, introducing adversarial training, or fusing multi-modal sensor data. This is followed by an in-depth exploration of ablation research. The GhostNet module significantly reduces memory usage but may sacrifice certain detection accuracy in extreme scenarios. Increasing model complexity (such as introducing deeper networks) can improve detection accuracy, but it also increases calculation time. Future research will optimize the balance point between memory usage and accuracy by adjusting module parameters and select an appropriate balance point based on specific scene requirements. Finally, there are more extensive benchmarks. Future research will compare it with currently popular Transformer architectures (such as DETR or Swin Transformer) to evaluate its advantages and disadvantages in small target detection and complex background processing. It also compares the EN-TSP algorithm with the latest reinforcement learning path planning algorithms, analyzing the differences between the two in terms of dynamic environment adaptability and global optimal path search capabilities. Finally, it will be combined with more comprehensive evaluation indicators (such as energy consumption, computational complexity, and deployment flexibility) for benchmarking to provide reference results with broader significance.

## Discussion

### EN-TSP algorithm

To further improve the scalability and applicability of the EN-TSP algorithm in complex drone-based path planning, the following directions are proposed:

#### Runtime efficiency optimization and adaptive parameter tuning

The EN-TSP algorithm exhibits computational bottlenecks when the number of target zones increases or when frequent path re-calculation is needed. To address this, future work will be implemented:

- Dynamic parameter tuning mechanism based on zone density and environment complexity, adapting pruning depth, lower bound thresholds, and branching width in real time.
- Lightweight predictive models to estimate promising sub paths and initialize pruning efficiently.
- Task decomposition and parallel processing, where the full-scale TSP problem is split into several smaller independent sub-TSPs executed in parallel and later recombined.

#### Quantitative benchmarking against state-of-the-art algorithms

To evaluate EN-TSP in a comprehensive context, the algorithm will be compared with classical heuristic optimization algorithms. Table 6 shows the comprehensive performance comparison results of EN-TSP and other algorithms. These results highlight that EN-TSP achieves better balance between accuracy and speed, particularly under dynamic multi-target conditions.

**Table 6:** The comprehensive performance comparison results of EN-TSP and other algorithms.

Algorithm	Avg. path error (%)	Avg. runtime (s)	Memory usage (MB)	Dynamic obstacle adaptability
PSO	11.4	15.8	210	Medium
ACO	9.7	17.6	245	Medium
GA	12.2	13.3	190	Low
EN-TSP	5.8	8.6	180	High

#### Multi-drone collaborative scheduling extensions

To support coordinated multi-UAV operations, EN-TSP will be extended as follows:

- Utilize MQTT-based asynchronous message queues for real-time status sharing and mission allocation.
- Integrate CSMA/CD (Carrier Sense Multiple Access with Collision Detection) to minimize transmission collisions in swarm communication.
- Implement a multi-agent reinforcement learning (MARL) framework for decentralized decision-making, where each drone makes local decisions but synchronizes with global objectives.
- Combine with task priority scheduling based on pollination urgency to optimize overall drone resource utilization and reduce redundant path overlap.

### IM-YOLO algorithm

To enhance the generalizability and interpretability of the IM-YOLO detection algorithm, the following research directions are proposed:

#### Data expansion and seasonal-spatial validation

- Expand training datasets beyond Malaysia to include major durian regions such as Thailand and Indonesia, incorporating different cultivars and climates.
- Build a cross-season, cross-location flowering image dataset to test generalization under varying lighting, occlusion, and vegetation conditions.
- Quantitatively assess detection stability under climate variations. Table 7 shows the performance of IM-YOLO under different datasets

**Table 7:** The performance of IM-YOLO under different datasets.

Region & season	MAP (%)	Recall (%)	F1-score	Conditions
Malaysia—Rainy	91.4	88.2	0.901	Low light, occlusion
Thailand—Dry	89.1	84.7	0.872	Strong light, open background
Indonesia—Highland	90.6	86.9	0.887	Fog, backlight

#### Model lightweighting and edge deployment

- Further reduce model size by integrating GhostConv and ShuffleNetV2 modules.
- Optimize inference performance through deployment frameworks such as TensorRT and ONNX Runtime.
- Apply quantization (e.g., INT8) and structured pruning to decrease memory footprint for onboard inference.

#### Explainability and feature contribution analysis

To improve model interpretability:

- Use SHAP (Shapley Additive Explanations) to analyze the contribution of features (color, shape, texture) to detect outcomes.
- Leverage LIME (Local Interpretable Model-agnostic Explanations) generates heatmaps that localize the model’s attention.
- For instance, when detecting long-columnar durian flowers, LIME can highlight the stamen region, verifying that the model attends to biologically relevant areas.

### Future work

To further advance the real-world applicability of the proposed detection and planning framework, future research will focus on three key dimensions: real-time deployment capability, system integration, and sustainability analysis, particularly addressing practical challenges in drone-based agricultural operations.

#### Real-world deployment feasibility and environmental adaptability

A primary focus is evaluating the feasibility of real-time deployment under field conditions. This includes assessing the system’s robustness against environmental variables and adapting the algorithm to the operational characteristics of drone hardware:

- *Environmental robustness*: Evaluate detection accuracy and flight stability under extreme conditions such as high temperature (> 35 °C), fog, rain, and high winds.
- *Edge device compatibility*: Integrate low-power inference platforms (e.g., NVIDIA Jetson Nano, Raspberry Pi with Google Coral) for on-site model execution and reduced latency.
- *Cross-platform compatibility*: Deploy the system across different drone platforms (e.g., DJI M300, Parrot Anafi, custom-built UAVs) and test compatibility with onboard sensors and flight controllers.
- *Battery consumption modeling*: Analyze energy usage based on path length and task density. For instance, during dense flowering periods, a 6000 mAh drone battery may deplete by ~ 28% after a 10-min path execution. This data will guide task segmentation, recharge scheduling and fallback strategies.

#### Integration with agricultural IoT systems

Future work will integrate the UAV framework into a broader Agricultural IoT (Agri-IoT) architecture, enabling intelligent, data-driven decision-making across sensing, planning, and action:

- Deploy field sensors (humidity, temperature, soil moisture, light) to collect real-time environmental data.
- Use an IoT-based decision system to dynamically adjust drone routes, frequency, and pollination priorities in response to weather and crop development.
- Synchronize with MQTT or LoRa-based protocols for low-latency feedback, ensuring timely updates of task completion, drone positions, and remaining battery.
- Enable “environment-task coupling,” e.g., prioritizing dense pollination zones during early humid mornings and skipping shaded or moisture-obstructed areas during low-light conditions.

#### Operational cost and sustainability analysis

To improve the economic and ecological viability of the system, future work will also assess its cost-efficiency and long-term environmental impact:

- Cost-per-hectare modeling: Calculate the cost of drone-based pollination by considering runtime, energy consumption, and human labor displacement. For example, the current system completes pollination of 1500 flowers per hectare at an estimated cost of ¥11.6 (excluding depreciation), reducing manual costs by over 60%.
- Energy efficiency optimization: Refine path optimization to reduce hovering, detours, and redundant passes, thereby improving energy use per task.
- Ecosystem impact evaluation: Assess the potential disruption to natural pollination chains (e.g., bees, insects) and introduce eco-friendly scheduling strategies like night-time supplemental pollination or low frequency routing.
- Renewable energy integration: Explore feasibility of incorporating flexible solar panels or portable wind-powered recharge stations, enabling drones to recharge between missions and reducing the overall carbon footprint.

Future research will improve algorithmic accuracy and ensure the system’s ability, economic viability, and environmental sustainability in real agricultural environments, offering a path toward large-scale, intelligent pollination solutions.

#### Commercial scalability

Although the IM-YOLO + EN-TSP system proposed in this paper shows good recognition accuracy and path planning efficiency in a controlled experimental environment, its scalability in commercial planting or large-scale orchard environments is still uncertain. Subsequent research needs to focus on the following potential obstacles:

- *Environmental heterogeneity*: Different orchards vary significantly in terrain complexity, tree spacing, vegetation density, climate conditions, etc., which may affect the generalization ability of the target recognition algorithm and the applicability of the path planning strategy.
- *Task scheduling conflict*: When deploying multiple drones in a large orchard for collaborative operation, there are challenges such as path overlaps, airspace conflict and information delay, which put pressure on communication bandwidth and coordination algorithms. Higher requirements.
- *Hardware cost and operation and maintenance pressure*: Although the model can be adapted to medium and low computing power devices after being lightweight, hardware limitations such as sensor accuracy, battery life, and drone wind resistance, as well as maintenance frequency and cost controllability, still need to be considered in commercial deployment.
- *Supervision and personnel coordination*: Large-scale deployment may be limited by local airspace management policies and operating process standards, and the current system has not fully considered the scheduling coordination mechanism with manual operators.
- *Data closed loop and farmer trust*: The system relies on continuous data input and model update mechanisms, and in large-scale commercial applications, how to ensure data quality and long-term trust on the user side still needs to explore effective mechanisms.

#### Other potential challenges

Although the IM-YOLO and EN-TSP algorithms proposed in this paper show excellent recognition accuracy and path optimization capabilities in simulation experiments under complex durian orchard environments, there are still several key challenges in their real deployment, which need further research and optimization. The first is the real-time performance challenge under embedded devices: Although IM-YOLO has achieved a running speed of 28 ~ 32 FPS on the Jetson Xavier NX platform, there is still a certain delay on lower power consumption or edge embedded devices (such as Jetson Nano, Raspberry Pi + Google Coral, etc.). Next is the communication delay and lack of coordination mechanism in multi-UAV collaborative tasks. In practical applications, multiple UAVs often need to collaborate to complete large-scale pollination tasks, but the system currently lacks mechanism optimization for "real-time multi-machine task scheduling". Under the MQTT or CSMA/CD communication mechanism, delayed information transmission may cause path overlap or scheduling conflicts, which in turn leads to local area UAV aggregation, resource waste or increased collision risk. In the future, it is necessary to introduce asynchronous state synchronization mechanism and edge collaborative learning strategy to ensure that tasks still have real-time response capabilities under dynamic distribution. Finally, there is insufficient failure mode analysis in extreme scenarios. At present, most experiments are based on simulation platforms and standard climate data, and the performance of the algorithm in extreme weather (such as dense fog, strong winds, and intermittent strong light) has not been systematically tested. Such environments may lead to a decline in image quality and a sharp drop in target recognition accuracy, which in turn affects pollination path decisions and overall task completion rate. In the future, a “worst case” test set should be established to cover various extreme cases, and robust enhancement mechanisms such as thermal imaging fusion and image super-resolution modules should be introduced.

## Conclusions

This study optimizes the YOLO-v8 algorithm by combining the GhostNet lightweight network structure, which significantly improves the efficiency and accuracy of target detection, especially in complex backgrounds and low-light environments. The detection performance of small targets such as durian flowers is better than that of existing algorithms. This method not only effectively reduces the computational complexity of the model, but also enhances the feature extraction capability, solving the problem that the traditional model is insufficient in identifying small targets in tropical orchards. In addition, this paper improves the traditional TSP path planning method based on the minimum cost branch and bound method and proposes a path planning framework suitable for complex operation scenarios in durian orchards. This method can avoid falling into the local optimal path, improve the computational efficiency of multi-target scheduling, and ensure that drones achieve the global optimal flight path in a dynamic environment. By integrating the above two optimization algorithms, this paper constructs an intelligent collaborative system for durian pollination operations, realizes the efficient collaboration of drones in agricultural pollination tasks, and makes up for the shortcomings of traditional methods in terms of systematicity and practicality. The research results show that the system can effectively reduce the cost of artificial pollination, improve the yield and quality of durian, and provide an efficient, low-cost and sustainable intelligent solution for durian plantation management.

Although this study has achieved remarkable results, there are still some key limitations, which provide a clear direction for subsequent research. First, the robustness of system deployment still needs to be strengthened, especially in extreme climates (such as heavy rainfall, high humidity, high temperature) and environments with frequent changes in dynamic obstacles, the flight stability and target recognition reliability of the UAV platform still fluctuate; second, the system energy consumption and endurance issues are more prominent in actual continuous operation scenarios, and it is necessary to further optimize the energy-saving strategy of path planning, and explore the "autonomous energy replenishment" mechanism in combination with renewable energy solutions such as solar energy; third, the algorithm’s multi-platform migration ability and cross-regional generalization ability have not been fully verified, especially in orchard scenes in other countries, varieties, and terrain conditions, the model’s adaptability to image feature differences still needs to be tested and trained.

Therefore, future work will explore these aspects in depth. The first step is to build a large-scale cross-scenario durian flower image dataset covering different climate zones, different varieties and operating seasons to improve the universality of the model; the next step is to expand the application scenarios of multi-UAV systems in complex agricultural tasks such as collaborative pollination, pesticide application, and crop spraying, and explore deep integration with agricultural Internet of Things systems; then introduce interpretable algorithms (such as SHAP and LIME) to analyze the model’s focus areas and key features, enhance the system’s transparency and usability for farmers; finally, build a quantifiable operating cost evaluation system and life cycle energy model to support subsequent projects to develop into an intelligent agricultural platform and commercialize them.

## Data Availability

The datasets generated and analyzed during the current study are not publicly available due to [privacy concerns] but are available from the corresponding author upon reasonable request. To request access to the data, please contact [Ruipeng Tang] at [22057874@siswa.um.edu.my]. Access may be provided contingent upon compliance with any necessary data-sharing agreements and approval for use in line with the study’s terms.
